# Editorial: Purinergic signaling and neuroinflammation

**DOI:** 10.3389/fphar.2022.1113063

**Published:** 2022-12-15

**Authors:** Charles Elias Assmann, Savina Apolloni, Zuleide Maria Ignácio, Margarete Dulce Bagatini

**Affiliations:** ^1^ Department of Biochemistry and Molecular Biology, Federal University of Santa Maria (UFSM), Santa Maria, Rio Grande do Sul, Brazil; ^2^ Department of Biology, Tor Vergata University of Rome, Roma, Italy; ^3^ Laboratory of Physiology, Pharmacology and Psychopathology, Graduate Program in Biomedical Sciences, Federal University of Fronteira Sul, Chapecó, Santa Catarina, Brazil; ^4^ Laboratory of Cell Culture, Graduate Program in Biomedical Sciences, Federal University of Fronteira Sul, Chapecó, Santa Catarina, Brazil

**Keywords:** purinergic signaling, neuroinflammation, purinoceptors, immunopharmacology, neuropharmacology

Severe and chronic neuroinflammation is a pathological process that can culminate in apoptosis and necrosis, with synaptic damage and even neuronal loss. Among the events involved are the activation of glial cells, increased release of inflammatory cytokines, and damage to the blood-brain barrier (BBB), leading to increased BBB permeability. These events are involved in neurodegenerative pathologies and neuropsychiatric disorders (Beamer et al., 2016; Kölliker-Frers et al., 2021). The NLR family pyrin domain containing 3 (NLRP3) gene encodes NOD-, LRR- and pyrin domain-containing protein 3, constituting an inflammasome complex, which activates the caspase-1 protein, induces an increase in inflammatory cytokines and nuclear factor κB (NF-κB) activation, with a consequent increase in genetic transcription (Swanson et al., 2019).

Among the critical mechanisms involved in the molecular events that end up in neuroinflammation, the purinergic system plays a crucial role, considering its function in a wide range of underlying biological processes (Beamer et al., 2016). The purinergic system comprises adenosine triphosphate (ATP) and its metabolites, adenosine diphosphate (ADP), adenosine monophosphate (AMP), and adenosine. These molecules exert several physiological functions through a range of purinergic receptors classified as purinoceptors P1 and P2. The P1 purinoceptors are a family of four types of metabotropic G protein-coupled receptors (A1, A2A, A2B, and A3), whose functions are mediated by adenosine. The P2 purinoceptors are nucleotide receptors, better known as ATP receptors, and are subdivided into ionotropic receptors, P2X, with seven subtypes (P2X1-P2X7) and metabotropic receptors coupled to G protein, P2Y, with eight subtypes (P2Y1, P2Y2, P2Y4, P2Y6, P2Y11-P2Y14) (Burnstock, 2020; Zarrinmayeh and Territo, 2020; Huang et al., 2021). Some of the P2Y receptors are responsive to the pyrimidines uridine 5’ triphosphate (UTP) and uridine diphosphate (UDP) (Burnstock, 2020).

Activation of the P2X7 receptor in monocytes and macrophages constitutes a potent signal for activating the NLRP3 inflammasome, inducing the release of pro-inflammatory cytokines of the IL-1 family (Martínez-Banaclocha and Pelegrín, 2020). P2X7 receptors are located in the central nervous system (CNS), preferably in microglial cells, and are stimulated by large concentrations of ATP released in various situations of injuries and neurodegenerative diseases (Illes et al., 2020). Through P2 receptors, ATP activates immune cells, inducing inflammation. Subsequently, ATP is metabolized by nucleotidase enzymes. Adenosine, one of the final metabolites of ATP, has an anti-inflammatory function through P1 receptors (Huang et al., 2021).

In this special Research Topic, an overview of the relationship between neuroinflammation and purinergic signaling has been highlighted by a mini-review and several original research papers. Two research articles in this Research Topic focused on the nociceptive transmission of inflammatory and neuroinflammatory pain mediated by purinergic receptors.

Qiao et al. demonstrated that the purinergic ionotropic receptor P2X3 is involved in neuroinflammatory conditions, providing a novel peripheral mechanism underlying pain sensitization. The P2X3 receptor is a target of lysophosphatidic acid (LPA), an endogenous lipid metabolite released during tissue injury or inflammation states, contributing to nociceptive behaviors. The Authors indeed demonstrated that LPA enhances the functional activity of P2X3 receptors. LPA enhanced α,β-meATP-evoked electrophysiological activity in rat dorsal root ganglia (DRG) neurons and exacerbated α,β-meATP-induced nociceptive behaviors in rats. LPA may increase P2X3 receptor-mediated currents and action potential bursts by sensitizing co-existed P2X3 receptors located on the nociceptive sensory terminals, resulting in exacerbated nociceptive behaviors in rats. The enhancement of the P2X3 receptor by LPA may have pathophysiological significance. Under pathological conditions, such as inflammation, both LPA and ATP signaling may appear together. Peripherally, ATP can be released from inflammatory cells as an “injury” signaling, resulting in a nociceptive response by directly activating P2X3-containing receptors in nociceptors.

In the research article by Peng et al., the Authors point to the role of the P2Y12 metabotropic receptor in hyperalgesia. This study showed that in the DRG of the human immunodeficiency virus (HIV) glycoprotein 120 (gp120) rats, a model of neuroinflammatory pain, P2Y12 mRNA and protein are significantly increased. In contrast, treatment with small interfering RNA that targets the long non-coding RNA uc.48+ reduces P2Y12 expression and function in DRG neurons, accompanied by the decreased activation of p38 MAPK in gp120 rats. Remarkably, uc.48+ silencing markedly mitigates gp120-induced hyperalgesia. The Authors propose that neuronal P2Y12 receptors might be involved in HIV gp120-induced neuroinflammatory pain, suggesting that targeting uc48+ by downregulating P2Y12 could be an efficient strategy for neuroinflammatory diseases, hampering both mechanical and thermal hyperalgesia.

The article by Chai et al. indicated that NLRP3-mediated pyroptosis exerted an essential effect on depressive symptoms. Precisely, salidroside can ameliorate pyroptosis by suppressing the P2X7/NF-κB/NLRP3 pathway. Furthermore, they revealed that salidroside provides novel therapeutic strategies for the treatment of depression. Depression is a common and serious mental disorder. Therefore, this study provides new insights into the potential treatment options for depression.

Finally, in the Review Article, Nobili et al. studied the therapeutic potential of astrocyte purinergic signaling in epilepsy and multiple sclerosis. Epilepsy and multiple sclerosis, two of the most common neurological diseases, are characterized by the establishment of an inflammatory environment in the central nervous system that drives disease progression and impacts neurodegeneration. Current therapeutic approaches in the treatment of epilepsy and multiple sclerosis are targeting neuronal activity and immune cell response, respectively. However, the lack of fully efficient responses to the available treatments obviously shows the need to search for novel therapeutic candidates that will not exclusively target neurons or immune cells. ATP release and astroglial-mediated purinergic signaling are crucial mechanisms through which astrocytes communicate between themselves, with neurons in epilepsy and with autoreactive immune cells, making them suitable targets for designing innovative disease-modifying strategies. In the case of epilepsy, this would mean developing effective therapies that will not just target neurons, but also astrocytes, which by themselves provide conditions for proper neuronal functioning. Similarly in multiple sclerosis, demyelination and neuronal loss could be rescued by effectively controlling and targeting astrocyte interaction with autoreactive immune cells.

The collection of articles included in this Research Topic provides evidence of the interplay between neuroinflammation and purinergic signaling. Also, the papers in this Research Topic give insights into the paramount and complex involvement of purinergic system components in the inflammatory events in various disease conditions, such as depression, epilepsy, and multiple sclerosis, among others. Taken together, the articles in this Research Topic suggest the purinergic system as a main signaling pathway to be further investigated to understand its implications in the inflammatory reactions associated with different pathological scenarios and as a possible target for the development of new and effective pharmacological therapies.
